# Does Total Knee Arthroplasty Positively Affect Body Static-Dynamic Balance and Fall Risk Parameters in Patients With Satisfactory Functional Scores?

**DOI:** 10.7759/cureus.30207

**Published:** 2022-10-12

**Authors:** Serkan Davut, Irem Huzmeli, Hasan Hallaceli, Aydıner Kalacı

**Affiliations:** 1 Department of Orthopedics and Traumatology, Tayfur Ata Sokmen Faculty of Medicine, Hatay Mustafa Kemal University, Hatay, TUR; 2 Department of Physiotherapy and Rehabilitation, Faculty of Health Sciences, Hatay Mustafa Kemal University, Hatay, TUR

**Keywords:** prevention, falls, balance, total knee arthroplasty, osteoarthritis

## Abstract

Objective

The aim of this study was to determine the balance problems and risk of falling by using digital or computerized methods in patients who underwent total knee arthroplasty (TKA) and have satisfactory functional scores in the early postoperative period.

Methodology

A total of 31 participants (24 women, seven men; mean age: 61.93 ±10.75 years; range: 49-82 years) who underwent unilateral TKA were included. The fall risk was evaluated using the time up-and-go (TUG) test and computerized platforms. Patient-reported pain, stiffness, and physical functional outcome measures [Western Ontario and McMaster Universities Osteoarthritis Index (WOMAC) and Oxford Knee Score (OKS)] and posture (New York Posture Rating Chart) were evaluated.

Results

Based on the WOMAC scores, there was a significant impact on self-reported pain (p˂0.001), function (p=0.001), and stiffness (p=0.001) between preoperative and postoperative results. The OKS (p=0.006) and the TUG score (p=0.004) improved significantly, but the posture scores remained the same after the surgery. There was a statistically significant difference between the preoperative and third-month postoperative test results of the stabilometric test, bipedal opened eye, bipedal closed eye, monopedal right, and monopedal left foot static balance tests (p˂0.05). However, the disequilibrium and equilibrium dynamic balance values ​​remained unchanged three months after TKA.

Conclusions

Satisfactory functional scores according to WOMAC or OKS were achieved in the early postoperative period. However, posture and dynamic balance problems related to falling risk continued to persist in the same period. Although the TUG test results were statistically significant, they also showed fall risk values. Fall risk and postural problems should be analyzed objectively using computerized methods. Early rehabilitation programs after TKA in elderly individuals should be designed accordingly and close attention must be paid to fall risks.

## Introduction

Osteoarthritis (OA) causes joint pain, limitation of joint movements, trunk postural stability problems, and finally, loss of function [1,2]. Total knee arthroplasty (TKA) relieves the symptoms of pain associated with severe OA, corrects the deformity, and ultimately provides functional facilitation [3,4]. The rate of primary TKA is expected to continue to increase exponentially in the coming years [5]. The main aim after TKA is to achieve not only painless joint movement but also a high level of activity along with a high quality of life. Athletic or high performance is sought even at an advanced age, and being ambulatory alone is no longer considered sufficient by many patients [6]. Quality of life depends on preoperative status, comorbidity, sociocultural-habitual activities, and many physical parameters including balance and postural control [7]. Some studies have reported that up to 30% of patients are dissatisfied five years after surgery [6]. At the same time, only a few studies have reported a higher incidence of falls after TKA compared to asymptomatic healthy older people, ranging from 17% to 48% [7,8].

Postural stability is important for controlling the position of the body and maintaining static and dynamic balance during activities such as walking, rising from a bed, getting in/out of a bath, and performing domestic tasks. Static balance maintains balance while standing at one point, while dynamic balance provides balance during movement [9]. Postural stability impairment is one of the main causes of falls in the elderly and is therefore an important public health problem in patients with OA [10].

Postural stability impairment develops as a result of proprioceptive deficiency, muscle weakness, and knee pain in patients with knee OA [11]. The relationship between trunk balance and postural stability parameters after TKA has recently been considered by researchers [11,12]. The static and dynamic balance tests of the human body, also known as stability tests, are conducted using field tests, which can be evaluated as either static or dynamic. The static balance is evaluated by a single leg stance test, while the dynamic balance is assessed by the time up-and-go test (TUG) [11,13-15]. In addition, the balance of the body can be objectively evaluated with physiological profile assessment and digital or computerized platforms [16-19].

The prevalence of falls is expected to decrease as a result of the clinical outcome of TKA, with relieving pain, satisfactory function, and enhanced proprioception in the postoperative period [20,21]. In contrast, some studies have indicated that sacrificing the anterior cruciate ligament creates proprioceptive/balance deficits after TKA, resulting in an increased risk of falling [22]. Even so, there are few evidence-based studies that show the results of clinical situations associated with the postural change of the trunk and static and dynamic balance problems of the body in the early period after TKA [11,13,16,23].

To date, there are no data on the evaluation of static and dynamic balance problems, and consequently on the fall risk, with objective or computerized methods in patients with TKA who reported good functional results in the short term. These data are important for fall prevention, and rehabilitation programs should be planned in the early period after surgery. The aim of this study was to determine the balance problems and risk of falling with computerized platform methods in patients who underwent TKA and have satisfactory functional scores in the early postoperative period.

## Materials and methods

Ethical approval and consent

This study was approved by the Ethics Committee of the Medical Faculty of Hatay Mustafa Kemal University (approval no/date: 2018-118/June 28, 2018) and conducted according to the tenets of the Declaration of Helsinki. All patients were informed before the study about the study details, and a written informed consent form was signed by all patients.

Participants

The study included patients between the ages of 49 and 82 years who underwent unilateral TKA between July 1, 2018, and May 31, 2019. All patients were evaluated preoperatively and postoperatively. The inclusion criteria were as follows: patients with OA symptoms scheduled for primary total TKA, with no balance or gait disturbance. The exclusion criteria were as follows: patients with a history of musculoskeletal injury, history of any rheumatic disease, vestibular limitation affecting the balance, any neurologic disease leading to gait disturbance, and postoperative complications of the surgery, such as deep venous thrombosis or postoperative infection.

Study design

Clinical evaluations of all patients were conducted by two experienced orthopedics and traumatology specialists. All patients underwent surgery via the subvastus approach and a posterior stabilized total knee prosthesis design (Nexgen-Legacy, Zimmer Orthopedics, Warsaw, IN) was used. Functional status, pain, posture, and fall risk tests were evaluated twice by a physiotherapist before and after the surgery in all patients. A postoperative rehabilitation program was provided to all patients. The patients received approximately 30 minutes of a daily rehabilitation program, which included soft tissue therapy, active and strengthening exercises, and the facilitation of proper transfer techniques for mobility. This rehabilitation program included strengthening and walking training, which was revised as a home exercise.

Risk of fall

I. Static and Dynamic Balance

The risk of falling was analyzed by stabilometric and dynamic tests. The tests were carried out on two different platforms with the Techno Body Easy Prokin Portative Device (PK 200-Prokin System, Bergamo, Italy). After calibrating the instruments, patients were asked to place their bare feet on the static platform. The balance of the body in the ellipse area, perimeter, anteroposterior and mediolateral directions, in a standing upright position with computerized methods, was calculated digitally in mm or graphically [16,17]. The stabilometric test was evaluated with eyes open or closed on both legs and one leg. The dynamic test entails the evaluation of the instantaneous change in the body’s center of gravity on a moving platform on two legs. Three valid trials over 30 seconds were recorded after a 30-second trial, with a two to four-minute break between trials.

**Figure 1:**
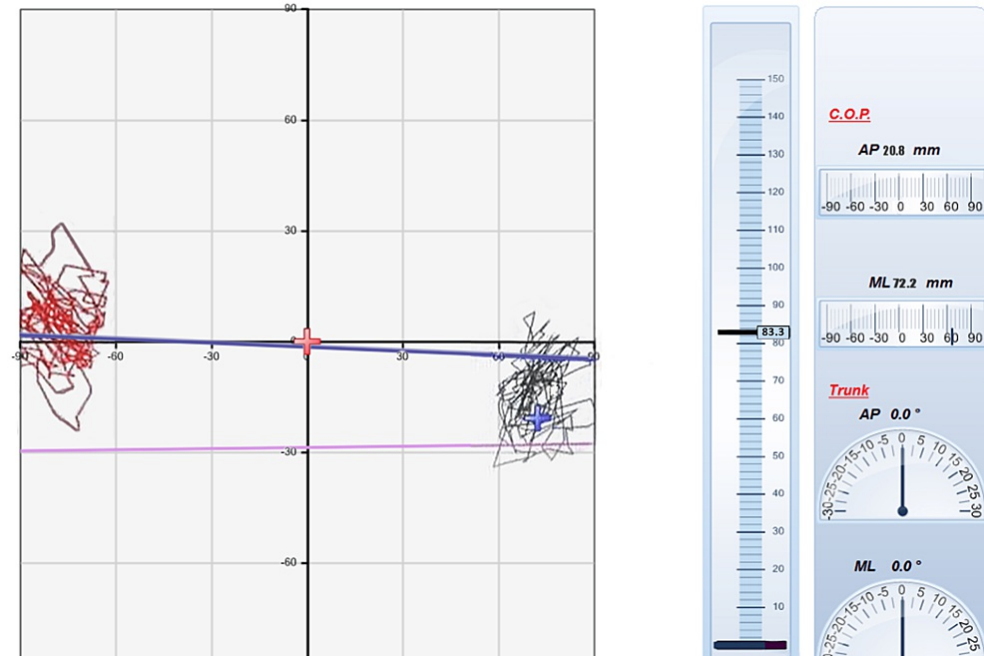
Preoperative drawings of a case's eyes-open stabilometric balance test (single leg stance balance test) The red-colored drawings show the balance data on the left, and the black-colored drawings show the right lower extremity C.O.P.: center of pressure; AP: anteroposterior; ML: medial-lateral

**Figure 2:**
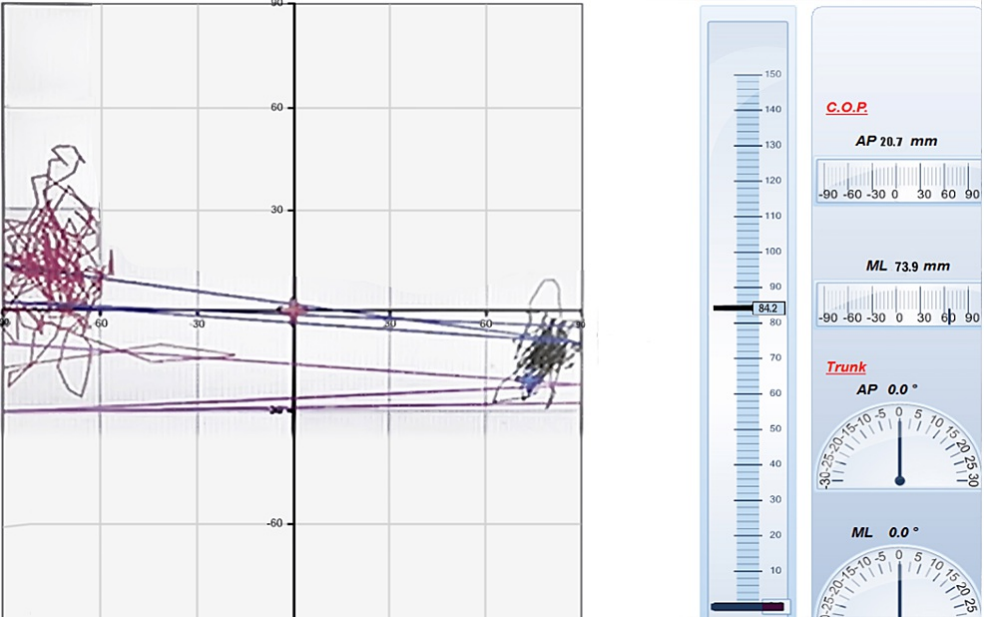
Postoperative drawings of a case's eyes-open stabilometric balance test (single leg stance balance test) The red-colored drawings show the balance data on the left, and the black-colored drawings show the right lower extremity C.O.P.: center of pressure; AP: anteroposterior; ML: medial-lateral

Static balance results were calculated using the following data of ellipse area (mm^2^) and were categorized as follows: 1-142.7 mm^2^: athletic, 142.8-215 mm^2^: normal, 215.1-284.5 mm^2^: poor (Figures 1, 2) [24].

**Figure 3:**
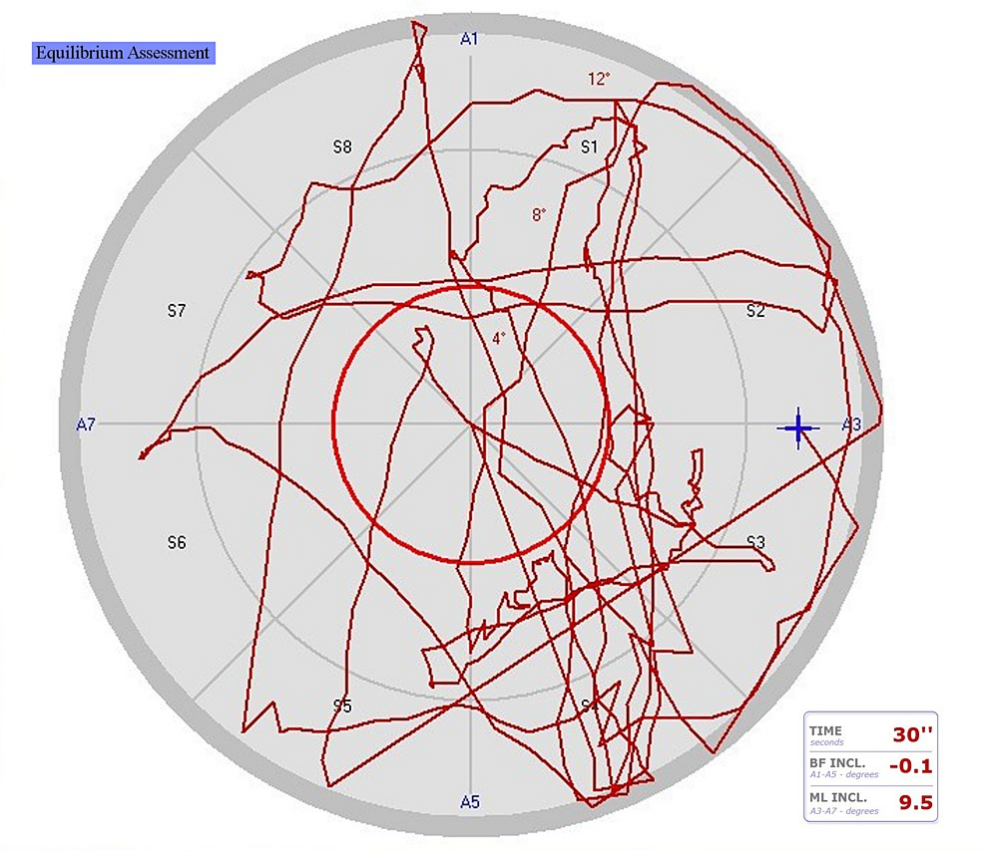
Preoperative drawings of a case's bipedal eyes-open equilibrium test BF INCL: backward-forward inclination; ML INCL: medial-lateral inclination; S: the tilting boards' surface areas (S1, S2, S3, S4, S5, S6, S7, and S8); A: axes (A1-A5=backward-forward, A3-A7=medial-lateral)

**Figure 4:**
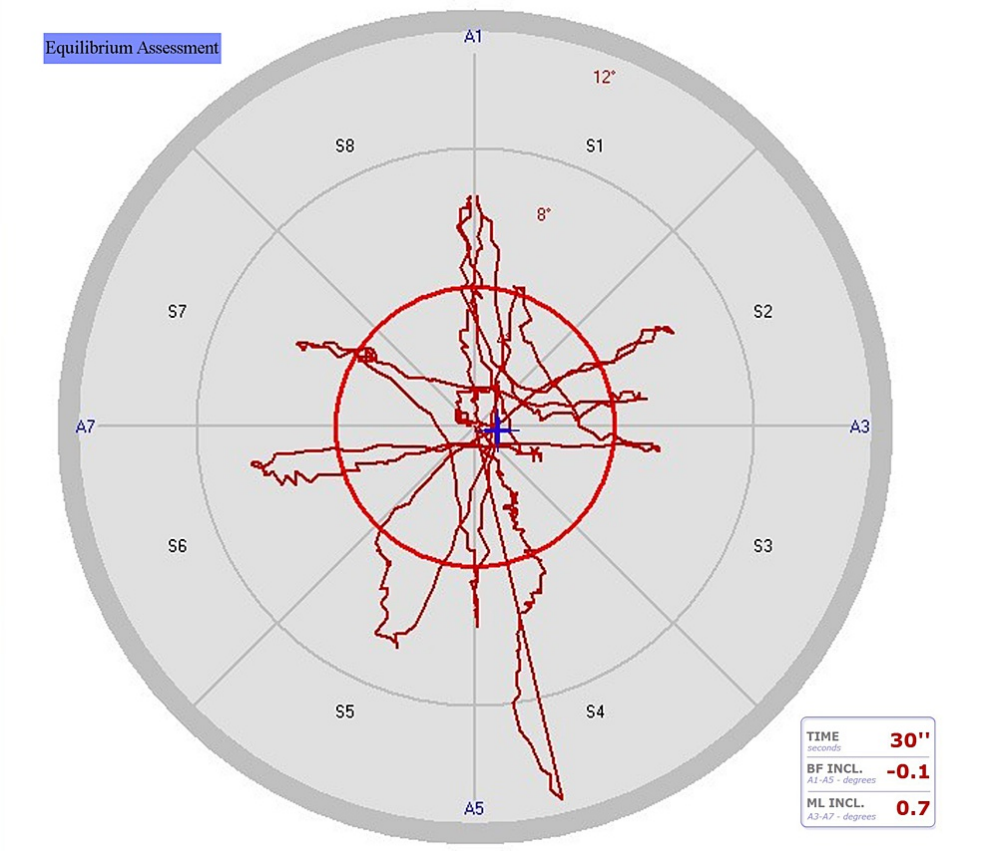
Postoperative drawings of a case's bipedal eyes-open equilibrium test The total area scanned (red scanned areas) in Figure 4 is less than that in Figure 3, indicating the changing balance situation BF INCL: backward-forward inclination; ML INCL: medial-lateral inclination; S: the tilting boards' surface areas (S1, S2, S3, S4, S5, S6, S7, and S8); A: axes (A1-A5=backward-forward, A3-A7=medial-lateral)

Dynamic equilibrium balance (Figures 3, 4) was assessed by the perimeter length, area gap percentage, medium speed, medium equilibrium center (medial-lateral), and medium equilibrium center (anteroposterior). Disequilibrium dynamic balance tests included the front/right standard deviation, back/left standard deviation, and distance medium error (%) [24].

II. The TUG Test

The test was analyzed as follows: the time that the person got up, walked, and sat again was recorded at a distance of three meters from the chair where the person is sitting. The test result was obtained using a stopwatch that was precisely measured to the nearest 1/100th of a second. The starting position, sitting posture, back support, and positioning of the upper and lower extremities of the test were standardized for each participant. Testing period times longer than 12 seconds indicated a risk of falling and functionality problems [25].

III: Functionality

The functional levels and features of the patients were evaluated using Western Ontario and McMaster Universities Osteoarthritis Index (WOMAC) outcome measures; this index assesses pain, stiffness, physical, social function, and emotional function and is scored between 0 and 54, with high scores indicating increased pain, stiffness, and loss of physical function. The Oxford Knee Score (OKS) is used to assess joint pain and physical function. The patients were asked 12 questions, and the overall score ranges from 0 (worst) to 48 (best). Both WOMAC and OKS are popular, reliable, valid, and sensitive. The Turkish version of OKS and WOMAC scores were used [26].

IV. Posture Analysis

Posture changes may occur in 13 different parts of the body, including all four extremities and the spine. Body posture was assessed in the lateral and posterior aspects and was scored by monitoring using the New York Posture Rating Chart (NYPRC). Accordingly, if the person showed correct posture, 5 points are given; 3 points are given in cases of moderately impaired posture, and 1 point if the person is severely impaired. The maximum total score obtained was 65 and the minimum was 13. According to the evaluation criteria of the chart, those who scored ≥45, 40-44, 30-39, 20-29, and ≥19 points were ranked as very good, good, medium, poor, and bad, respectively [27].

Statistical analysis

The “G*Power” package software program (G*Power, Version 3.1.9.4, Franz Faul, Universität Kiel, Germany) was used to calculate the required sample size for the study. According to a previous study [28], a calculation to obtain a sample consisting of 30 subjects was needed to obtain 90% power (1-β error probability), with effect size d=0.5673077, α=0.05 type I error, and β=0.10 type II error.

The research data were evaluated using SPSS Statistics for Windows, Version 20.0 (IBM Corp., Armonk, NY). Frequency distributions, arithmetic mean and standard deviation and median values were recorded in the evaluation of demographic and clinical characteristics. Compliance of variables to normal distribution was analyzed using visual and analytical methods (Kolmogorov-Smirnov/Shapiro-Wilk tests). Two related sample tests were used for in-group comparisons and paired sample t-tests were used as an alternative. The significance level was set at p<0.05.

## Results

**Figure 5:**
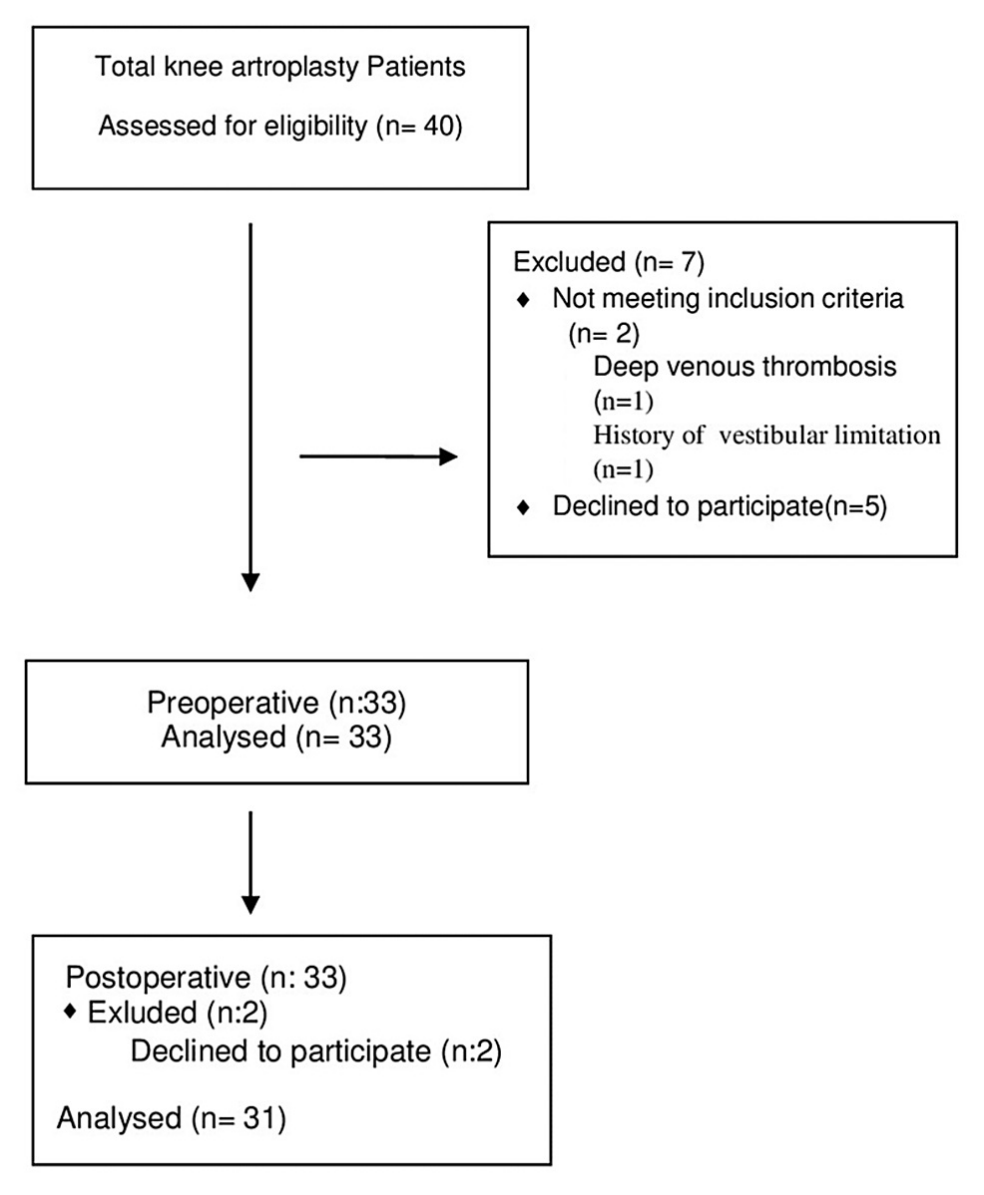
Flow diagram depicting the selection of patients with TKA TKA: total knee arthroplasty

A total of 40 patients with knee OA who underwent unilateral knee arthroplasty were enrolled in the present study. Of those, seven did not meet the inclusion criteria, and two refused to participate. Thus, 31 patients were ultimately recruited and analyzed (Figure 5).

The mean age of the 24 females (77.4%) was 63.54 ±7.40 years, while the mean age of the seven (22.6%) males was 56.42 ±17.96 years. The baseline demographic and clinical characteristics are shown in Table 1.

**Table 1:** The baseline demographic and clinical characteristics SD: standard deviation

Variables (n=31)	Values
Age, years, mean ±SD	61.93 ±10.75
Weight, kg, mean ±SD	84.91 ±12.76
Height, cm, mean ±SD	166.20 ±9.12
Body mass index, kg/m^2^,mean ±SD	30.94 ±5.53
Female, n (%)	24 (77.4)
Male, n (%)	7 (22.6)
Profession, n (%)	
Not working	19 (61.3)
Retired	6 (19.4)
Technician	2 (6.5)
Business manager	1 (3.2)
Other	3 (9.7)
Educational level, n (%)	
Tertiary	1 (3.2)
Secondary	14 (45.2)
Primary	12 (38.7)
None	4 (12.9)
Comorbidity, n (%)	
Cardiac arrhythmia	1 (3.2)
Hypertension	6 (19.4)
Diabetes mellitus	5 (16.1)
Neuropathy	1 (3.2)
Bronchial asthma	1 (3.2)
None	14 (45.2)
Knee arthroplasty side, n (%)	
Right	16 (51.6)
Left	15 (48.4)

**Figure 6:**
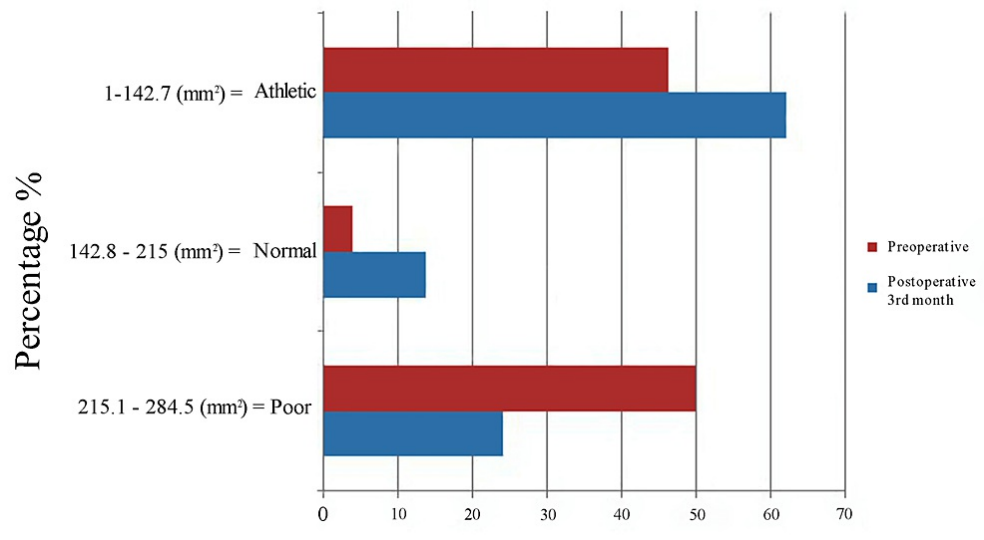
Changing rate of the static balance test results

**Figure 7:**
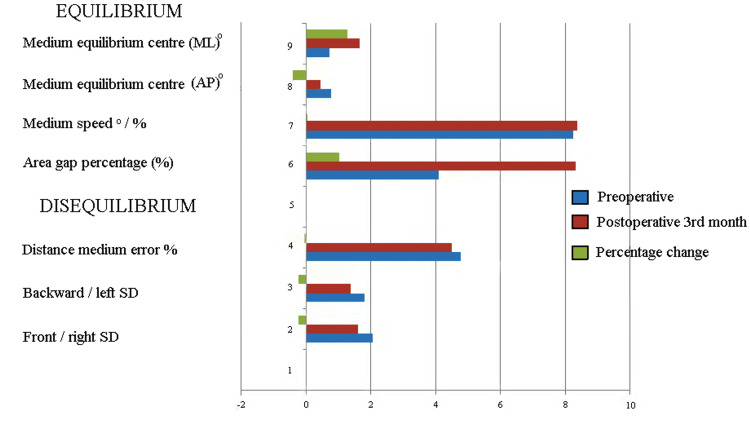
Changing rate of the dynamic balance test results SD: standard deviation; AP: anteroposterior; ML: medial-lateral

There was a statistically significant difference between the preoperative and third-month postoperative results of the stabilometric test, bipedal opened eye, bipedal closed eye, monopedal right, and monopedal left foot static balance tests (p˂0.05). However, there was no statistical significance in the disequilibrium and equilibrium dynamic balance results before and after surgery. Changes in the categorized static balance rates are shown in Figure 6 and Figure 7. The poor static balance rate changed from 50% to 24.1%, the normal balance rate changed from 3.8% to 13.8%, and the athletic balance changed from 46.2% to 62.1%.

According to the results shown in the graphs and Table 2, the dynamic values ​​remained unchanged three months after TKA.

**Table 2:** The results of the dynamic and static balance tests performed before and after surgery in the third month *P˂0.05 IQR: interquartile range; SD: standard deviation; z/t: z value calculated with Wilcoxon signed-rank test/t value calculated with paired samples test; FBSD: forward-backward standard deviation; MLSD: medium-lateral standard deviation; AP: anteroposterior; ML: medial-lateral

Variables	Preoperative	Postoperative third month		
			z/t	P-value
Static balance tests				
Stabilometric test				
Ellipse area, mm^2^, median (IQR)	168.14 (33.03-1063.10)	118.97 (39.40-211.72)	-2.40	0.016*
Perimeter, mm, median (IQR)	139.94 (91.34-346.52)	75.57 (49.48-115.84)	-3.32	0.001*
FBSD, median (IQR)	2.43 (1.21-5.79)	1.27 (0.94-1.78)	-2.71	0.007*
MLSD, median (IQR)	5.19 (1.91-10.24)	1.66 (1.32-5.84)	-2.32	0.020*
Bipedal opened eye				
Ellipse area, mm^2^, median (IQR)	167.49 (37.52-920.27)	31.06 (19.68-130.46)	-3.01	0.003*
Perimeter, mm, median (IQR)	155.96 (87.71-345.56)	68.34 (48.52-129.13)	-3.38	0.001*
FBSD, median (IQR)	1.79 (1.19-4.27)	1.01 (0.80-1.38)	-3.37	0.001*
MLSD, median (IQR)	4.52 (1.83-10.26)	1.72 (1.19-4.21)	-3.21	0.001*
Bipedal closed eye				
Ellipse area, mm^2^, median (IQR)	187.42 (44.77-1078.50)	41.74 (19.19-163.64)	-3.36	0.001*
Perimeter, mm, median (IQR)	235.61 (155.66-446.84)	88.73 (68.72-167.44)	-3.46	0.001*
FBSD, median (IQR)	2.20 (1.09-4.23)	1.27 (0.78-5.05)	-0.20	0.840
MLSD, median (IQR)	4.63 (1.90-11.66)	2.14 (1.36-8.17)	-2.39	0.017*
Monopedal right				
Ellipse area, mm^2^, median (IQR)	1805.90 (143.91-3379.20)	304.85 (116.01-1369.65)	-2.06	0.039*
Perimeter, mm, median (IQR)	391.93 (151.80-770.46)	179.47 (133.87-301.58)	-2.80	0.005*
FBSD, median (IQR)	12.49 (4.08-15.93)	5.28 (3.33-11.84)	-2.66	0.008*
MLSD, median (IQR)	7.35 (1.99-12.27)	4.14 (1.62-7.16)	-2.77	0.006*
Monopedal left				
Ellipse area, mm^2^, median (IQR)	1242.71 (126.18-2566.31)	198.05 (119.55-1289.90)	-2.61	0.009*
Perimeter, mm, median (IQR)	269.58 (129.30-609.06)	162.87 (123.74-304.02)	-2.66	0.008*
FBSD, median (IQR)	11.09 (3.17-16)	3.68 (3.04-9.09)	-2.66	0.008*
MLSD, median (IQR)	5.95 (2.26-9.26)	3.15 (2.10-7.01)	-2.31	0.021*
Dynamic balance test results				
Disequilibrium				
Front/right SD, median (IQR)	2.05 (1.4-2.91)	1.59 (0.83-2.20)	-1.82	0.067
Backward/left SD, median (IQR)	1.79 (0.95-3.48)	1.38 (0.91-2.31)	-1.79	0.073
Distance medium error %, mean ±SD	4.77 ±2.07	4.44 ±1.49	0.84	0.407
Equilibrium				
Perimeter length, mean ±SD	233.41 ±91.30	263.20 ±112.89	-1.46	0.154
Area gap percentage, median (IQR)	4.14 (0-19.69)	8.33 (1.32-17.03)	-0.64	0.518
Medium speed, %, mean ±SD	8.23 ±3.36	8.36 ±3.71	-0.20	0.841
Medium equilibrium center, AP, median (IQR)	0.78 (0.29-1.90)	0.45 (0-2.1)	-0.59	0.554
Medium equilibrium center, ML, median (IQR)	0.73 (0.23-2.36)	1.65 (0.25-3.40)	-1.94	0.052

Based on WOMAC scores, there was a significant effect on self-reported pain (p˂0.001), function (p=0.001), and stiffness (p=0.001). The WOMAC total score and WOMAC percentage were significantly different between the preoperative and postoperative results (p˂0.05). According to the OKS classification, the most common score was "poor" (62.5%) before surgery and "moderate" after surgery (37.5%). The OKS total (p=0.006) and OKS classification (p=0.047) improved after surgery. There was a significant decrease in the TUG test results (p=0.004) after surgery. However, the results of the NYPRC and classification were not statistically significant (Table 3).

**Table 3:** The functionality, fall risk, and posture test results *P˂0.05 SD: standard deviation; IQR: interquartile range; WOMAC: Western Ontario and McMaster Universities Osteoarthritis Index; TUG: time up-and-go test; NYPRS: New York Posture Rating score; NYPRCC: New York Posture Rating Chart classification; z/t/x^2^: z - Wilcoxon signed-ranks test value, t - paired samples test value, x^2 ^- chi-square test value

	Preoperative	Postoperative third-month	z/t/x^2^/p-value
	Functionality tests			
WOMAC total score, median (IQR)	64 (57-76)	31 (25.5-49.5)	-4.01/˂0.001*
WOMAC %, median (IQR)	66.66 (59.37-79.16)	32.29 (26.56-51.56)	-4.01/˂0.001*
WOMAC pain, mean ±SD	14.61 ±3.35	9.00 ±4.34	7.48/˂0.001*
WOMAC stiffness, median (IQR)	5 (4-6)	2 (2-4)	-3.34/0.001*
WOMAC function, mean ±SD	42.70 ±13.36	22.80 ±8.31	6.49/0.001*
Oxford total score, mean ±SD	17.95 ±10.88	26.76 ±9.19	-3.06/0.006*
Oxford classification, %			
Poor (0-19)	62.5	29.2	0.047*
Moderate (20-29)	20.8	37.5	
Good (30-39)	12.5	25.0	
Excellent (40-48)	4.2	8.3	
Fall risk test			
TUG, seconds, mean ±SD	18.62 ±6.16	16.36 ±5.38	3.20/0.004*
Body posture test			
NYPRS, mean ±SD	38.12 ±8.81	37.85 ±9.80	0.14/0.885*
NYPRCC, %			
Very good (˃45)	21.4	27.28	0.796*
Good (40-44)	0	5.6	
Middle (30-39)	57.1	44.4	
Not good (˂19)	21.4	22.2	

## Discussion

Neither the dynamic balance nor posture parameters improved significantly in the early postoperative period, which was related to fall risk. Although the TUG test results improved in a statistically significant manner, they showed a fall risk value. The most important finding of this study is that both TUG and dynamic balance tests are predictive of the risk of falling.

The balance-related fall mechanism in patients with OA has not been fully explained [8,9,20]. Balance deficits can affect the integrity of postural stability, and this is associated with an increased risk of falls and poor mobility in patients with OA [7,11,16]. Performing functional activities while maintaining balance is a complex function with both dynamic and static properties [8,17]. The risk of falling in elderly people with knee OA is associated with many parameters, including age, balance-related physiological capacity, joint degeneration, proprioceptive disorder, knee pain, and, particularly, quadriceps femoris weakness [7-9,17]. Very few studies have measured the progress of balance or fall risk in postoperative TKA patients according to the TUG test [11,13,15,29] and digital platforms [7,16,17,19]. Interestingly, some publications have reported a fall incidence of up to 30% after TKA, and have also mentioned that quadriceps femoris weakness is still a problem in the early period, especially in the first three [7,8] or six months [7].

The TUG and computerized balance tests are reliable and valid methods for evaluating both preoperative and postoperative periods [16,25]. The values of TUG change according to pathology and age parameters; a score of ≥12 or 14 seconds was accepted to indicate the risk of falling for geriatric patients with OA [25]. In some publications, statistically significant improvements were reported in OKS, WOMAC scores, and TUG test results at the end of the first postoperative year. However, these publications reported TUG scores >12 seconds, with improved functional scores [11,15,29]. Interestingly, Taniguchi et al. have reported that the risk of preoperative fall (upper 12 seconds) was found to disappear at the sixth postoperative month (below 12 seconds) according to the TUG, but the incidence of those who fell in this postoperative period was 26% in the same group [8]. Similarly, Swinkels et al. have reported a 22.7% fall incidence, in addition to good WOMAC scores and TUG values at six months postoperatively [29].

Functional scoring tests mostly comprise questions about pain, muscle strength, walking distance, stepping activity, use of transfer tools, and daily life activities [6,22]. Functional results according to the WOMAC and OKS parameters were similar to those reported in the literature [3]. Despite our patients demonstrating good functional results, it was observed that the dynamic balance of the trunk, which is required in many activities in daily life, was insufficient in the anterior, posterior, medial, and lateral directions. Using the stabilometric platform, it is possible to evaluate the change in time of the center of gravity of the trunk and its oscillations in the course of data uptake. The center of gravity of the trunk is directly related to the instantaneous postural changes [17,24]. The values of the displacement of the center of gravity of the trunk showed that there was no positive development in the postoperative period. In addition, according to the NYPRC, the shoulder, pelvis, thoracic, and lumbar regions had postural errors: the rate of moderate and poor posture in standing upright was 66.6%.

The TUG values detected in our study, similar to recent reports by some researchers, were observed to be >12 seconds, in addition to the presence of satisfactory functional scores in patients [11,13,15,29]. The TUG test determines the risk of falling based on the duration of walking [13,25]. However, with computerized platforms, measurements can provide more objective data on the balance change of the body on a moving platform [7,16,17]. In our study, we believe that the risk of falling persisted according to TUG, which has similar characteristic features in the literature and digital platform values.

Rehabilitation of posture and dynamic structures of the trunk is especially important in the geriatric population [13,16,27]. Chen et al. have stated that the postural index was associated with balance and postural stability, which is important in reducing the risk of falling in the elderly [23]. In contrast, TKA-related studies have advocated that there is no need for rehabilitation [6] or that home exercises alone are sufficient to generate good functional results at six, nine, and 12 months after surgery [3,11,15]. Similarly, these publications found satisfactory results with parameters such as functional capacity and quality of life indices [6,13,22]. Wang et al. have stated that 65.8% of patients with total knee prostheses could achieve a sufficient functional level with two-week home exercise programs [3]. However, Canovas et al. have reported that up to 30% of patients are dissatisfied five years after surgery [6]. Schwartz et al. and Moutzouri et al. have stated that the incidence of falls and deficits in proprioception may not be restored even one year after surgery in patients undergoing TKA [11,17]. When the related literature on functional outcomes was examined, the balance parameters were not considered. In addition, publications about fall risk in patients after TKA have been started to be noticed recently [7,8,16,17,29]. The current study showed no statistical difference between the presurgery status and postsurgery situation after three months, with a mid-range score for human body posture.

In addition to publications showing satisfactory functional results, the issue of dissatisfaction in the first five years and the risk of falling in the first six months are apparent. Computerized platforms measure the instability of the trunk in front-back and medial-lateral directions in detail. Thus, it conveys the patient's need to do exercises in the related directions more objectively than the TUG test.

We suggest that there is a need for rehabilitation programs focused on balance problems, not only according to WOMAC or OKS scores but also a computerized platform assessment. It is important for orthopedic clinics to determine the fall risk in the early period. We recommend that therapeutic exercise programs, including core stabilization, open-closed kinetic ring, and postural exercises, be implemented to improve the static and dynamic balance status, especially in the early postoperative period. To the best of our knowledge, this is a rare study that assesses the effect of TKA on static and dynamic balance and fall risk parameters in patients with satisfactory functional scores.

This study has some limitations. The TUG test and computerized balance system sub-results have not been compared in detail. In addition, the pain symptoms of the other knee with OA in the balanced state of the trunk have not been examined in detail. Comparisons should be made with studies examining different types of knee prosthesis designs and quadriceps femoris muscle strength of operated patients.

## Conclusions

Despite our patients demonstrating good functional results, it was observed that the dynamic balance of the trunk was insufficient in the anterior, posterior, medial, and lateral directions. Fall risk and postural problems should be analyzed objectively using computerized methods. Postural problems and dynamic balance problems persist after TKA. Rehabilitation programs for elderly individuals who undergo TKA should be designed and implemented to prevent falls.
